# Trap-Vaccinate-Release Program to Control Raccoon Rabies, New York, USA

**DOI:** 10.3201/eid1807.111485

**Published:** 2012-07

**Authors:** Sally Slavinski, Lee Humberg, Martin Lowney, Richard Simon, Neil Calvanese, Brooke Bregman, Daniel Kass, William Oleszko

**Affiliations:** New York City Department of Health and Mental Hygiene, New York, New York, USA (S. Slavinski, B. Bregman, D. Kass, W. Oleszko);; US Department of Agriculture, Rockville, Maryland, USA (L. Humberg, M. Lowney);; New York City Department of Parks and Recreation, New York (R. Simon);; and Central Park Conservancy, New York (N. Calvanese)

**Keywords:** rabies, raccoon, vaccination, epizootic, urban, New York, TVR, trap-vaccinate-release, viruses

## Abstract

In 2009, an outbreak of raccoon rabies in Central Park in New York City, New York, USA, infected 133 raccoons. Five persons and 2 dogs were exposed but did not become infected. A trap-vaccinate-release program vaccinated ≈500 raccoons and contributed to the end of the epizootic.

Central Park, described as an oasis in the midst of an urban jungle, spans 843 acres. Raccoons thrive in Central Park, an ideal habitat with an abundance of human refuse as food. Although not actually counted, the estimated raccoon population in the park is ≈500. Each year, Central Park receives >25 million visitors, offering ample opportunity for humans and off-leash dogs to be exposed to raccoons.

On August 27, 2009, a sick raccoon collected from Central Park in Manhattan tested positive for rabies virus, marking the emergence of an enzootic of raccoon rabies in Central Park. From December 2009 through December 2011, rabies test results for 133 raccoons collected in or near Central Park were also positive (Figure 1). The New York City Department of Health and Mental Hygiene (DOHMH) quickly assembled a task force with the objective of developing a response plan. The task force comprised members of the New York City DOHMH, the US Department of Agriculture Wildlife Services, the Central Park Conservancy, the New York City Department of Parks and Recreation, the New York State Department of Health, New York City Animal Care and Control, and the New York State Department of Environmental Conservation. A trap-vaccinate-release (TVR) plan was developed and implemented.

**Figure 1:**
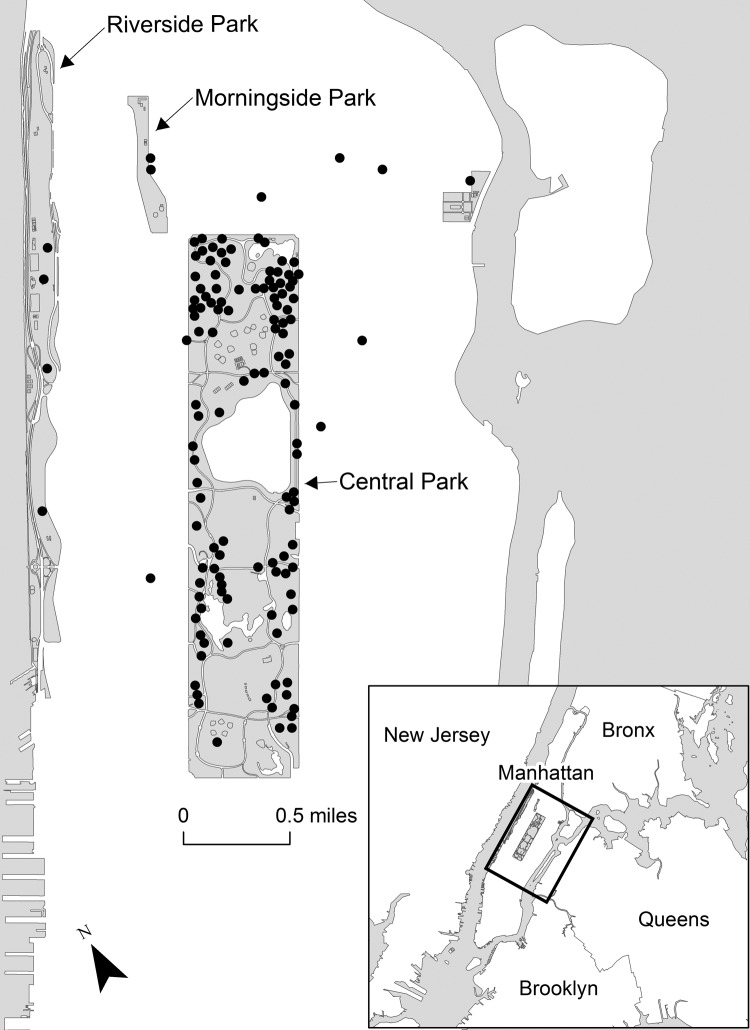
Location of rabid raccoons in and around Central Park, New York City, New York, USA, December 1, 2009–December 1, 2011. Each dot represents a rabid raccoon.

## The Program

The trap-vaccinate-release program goals were to reduce transmission of rabies among raccoons and prevent human and pet exposure to rabid raccoons. The few examples of raccoon rabies epizootics in similar settings often used a point infection control approach: oral rabies vaccine, depopulation of up to 80% of the raccoons, and TVR (*1**–**6*).

For the Central Park outbreak, oral rabies vaccine was ruled out because of the small but potential risk for vaccinia infections of humans (*7**,**8*), given the large volume of park visitors and poor raccoon seroconversion rates (9%–61%) (*9**–**14*). Depopulation was also eliminated because it would have overwhelmed the animal shelter system with demand for humane euthanasia and decapitations and because a national animal welfare organization and the public voiced opposition. Thus, the task force chose TVR.

The makeup of Central Park and the surrounding Manhattan area creates a fishbowl-style habitat; inside the park are acres of ideal living habitat, surrounded by a mass of concrete, roadways, vehicles, and pedestrians, to contain the raccoons. Central Park and 2 small parks in close proximity were targeted for 3 rounds of TVR. The public was notified through press releases, posters, flyers, electronic messaging, and the New York City 311 telephone information service. Community boards, political leaders, and human and animal health communities were notified directly. The DOHMH website was kept updated.

The 3 rounds of TVR were conducted: February 16–April 7, 2010, September 20–November 5, 2010, and November 28–December 16, 2011. Trapping efforts were focused in Central Park, followed by Morningside and Riverside Parks. Humane cage traps baited with marshmallows and anise oil as a scent attractant were placed in sites that were off limits or of limited access to the public and their dogs. Each trap had a rabies warning sign with emergency contact information.

Trapped raccoons were visually assessed for evidence of injury, illness, or death. Raccoons that were ill or injured were humanely euthanized and, along with those found dead, were submitted for rabies testing at the DOHMH Public Health Laboratory. Healthy raccoons were immobilized in the trap by a squeeze comb, given 1 mL of rabies vaccine in a thigh muscle, identified by placement of an ear tag, and then released at the capture site. Healthy, tagged raccoons that were later recaptured during the same round of TVR were released, but those recaptured during a subsequent TVR round were revaccinated.

During each round of TVR, 26–73 traps were set per night, resulting in 3,822 trap-nights (Table). A total of 1,129 raccoons were trapped (range 0–34/night), of which 484 raccoons were vaccinated and 112 were revaccinated. Among 232 raccoons, there were 586 instances of recapture (median 1, range 1–9).

**Table:** Results of raccoon trap-vaccinate-release program, New York, New York, USA, 2010

Round	Trap nights	No. trapped	No. vaccinated and released	No. recaptured	No. sick, injured, or dead
1	1,697	460	237	165	58
2	1,409	459*	148	307	1
3	716	210	99	114	1
Total	3,822	1,129	484	586	60

*Three raccoons were submitted for rabies testing after human exposures.

During round 1, of 460 raccoons trapped, 237 were deemed healthy, processed, and released (Table). Of 58 raccoons deemed unhealthy or found dead, 11 were rabies positive; of these, 0/6 were dead, 5/8 were sick, and 6/44 were injured. During round 2, of 459 raccoons trapped, 148 were newly vaccinated, tagged, and released, and 68 were revaccinated. One injured raccoon was euthanized; rabies test result was negative. None were found dead or sick. During round 3, of 210 raccoons trapped, 99 were newly vaccinated, tagged, and released, and 44 were revaccinated. One sick raccoon was euthanized; rabies test result was positive.

Among the vaccinated raccoons, rabies later developed in 14. The time between vaccination and recapture was 1–26 days (mean 10 days), suggesting that they were probably incubating the virus at the time of vaccination.

Exposures (confirmed or possible raccoon bites, contact with raccoon saliva) were identified for 5 persons and 2 dogs, all during January–June, 2010. Each person received rabies postexposure prophylaxis. Each dog was currently vaccinated against rabies, received a booster dose, and was monitored. Rabies did not develop in these persons or dogs.

**Figure 2:**
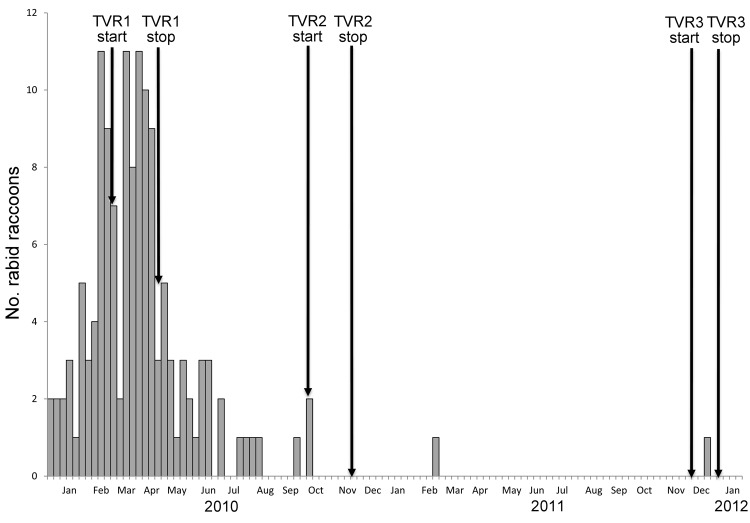
Number of rabid raccoons, Manhattan, New York, USA, by week, during and after the epizootic in Central Park and the corresponding dates of the 3 rounds of the trap-vaccinate-release program (TVR).

At the peak of the epizootic, 11 rabid raccoons were reported per week. By April, the epizootic started to decline (Figure 2), probably attributable to the natural depopulation resulting from rapid spread of the virus and to the population immunity resulting from TVR. The last cases of rabid raccoons were reported on February 2 and December 9, 2011.

## Conclusions

TVR seems to have effectively stemmed this epizootic of rabies in an established raccoon population. Critical to its success was the collaboration among federal, state, and local agencies and the private organizations responsible for park stewardship and animal control. This example suggests that a TVR program tailored to the geography, scope, and specifics of an epizootic in an urban area can successfully immunize a large population of raccoons and limit the potential for human and pet exposure to rabies virus.

Ongoing surveillance suggests that raccoon rabies has been successfully controlled in Manhattan. Had TVR not been implemented, the epizootic would probably have reached a state of continuous low-level enzootic activity. Given the natural border around Manhattan, it is unknown how rabies was initially introduced, but theories include illegal release of a raccoon or raccoon entry by bridge, tunnel, or even vehicle.

Annual use of TVR is not likely. However, after the immunized raccoon population declines and subsequent generations of susceptible animals predominate, another large epizootic could occur. Given the favorable park environment in which raccoon numbers can grow almost unchecked, population control should be explored as another way to prevent a recurrent epizootic with a similar explosive pattern. Public health and wildlife officials, along with academicians, should continue to explore efforts to develop safe, effective, and acceptable population control measures to help manage the unchecked growth of wildlife supported by urban centers.
